# Magneto-optical assessment of *Plasmodium* parasite growth via hemozoin crystal size

**DOI:** 10.1038/s41598-024-60988-6

**Published:** 2024-06-21

**Authors:** Ágnes Orbán, Jan-Jonas Schumacher, Szilvia Mucza, Ana Strinic, Petra Molnár, Réka Babai, András Halbritter, Beáta G. Vértessy, Stephan Karl, Stephan Krohns, István Kézsmárki

**Affiliations:** 1 Department of Physics, BME Budapest University of Technology and Economics, Budapest, 1111 Hungary; 2 Experimental Physics 5, Center for Electronic Correlations and Magnetism, Institute of Physics, University of Augsburg, 86159 Augsburg, Germany; 3 Malaria Research Laboratory, Institute of Enzymology, Research Centre for Natural Sciences, Budapest, 1117 Hungary; 4 Department of Applied Biotechnology and Food Sciences, BME Budapest University of Technology and Economics, Budapest, 1111 Hungary; 5 Vector-Borne Diseases Unit, PNG Institute of Medical Research, Madang, Madang Province 511 Papua New Guinea; 6 Australian Institute of Tropical Health and Medicine, James Cook University, Smithfield, QLS Australia

**Keywords:** Malaria, Hemozoin, Magnetic nanoparticles, Magneto-optical detection, Particle size distribution, Nanoscale biophysics, Diagnostic markers, Malaria

## Abstract

Hemozoin is a natural biomarker formed during the hemoglobin metabolism of *Plasmodium* parasites, the causative agents of malaria. The rotating-crystal magneto-optical detection (RMOD) has been developed for its rapid and sensitive detection both in cell cultures and patient samples. In the current article we demonstrate that, besides quantifying the overall concentration of hemozoin produced by the parasites, RMOD can also track the size distribution of the hemozoin crystals. We establish the relations between the magneto-optical signal, the mean parasite age and the median crystal size throughout one erythrocytic cycle of *Plasmodium falciparum* parasites, where the latter two are determined by optical and scanning electron microscopy, respectively. The significant correlation between the magneto-optical signal and the stage distribution of the parasites indicates that the RMOD method can be utilized for species-specific malaria diagnosis and for the quick assessment of drug efficacy.

## Introduction

After more than a decade of continuous decline since the millennium, malaria case numbers have stagnated in recent years, and the novel medical challenges facing the global community might further impede the pace of progress towards the control and elimination of this ancient disease^1^. Although efficient first-line treatments and long-established diagnostic methods are available, there is still a substantial degree of misdiagnosis of malaria due to resource limitations and certain shortcomings of the most widespread rapid diagnostic tests (RDTs), such as inadequate species-specificity and emergence of parasitic mutations lacking the targeted antigens^2–4^. Thus, the development of better diagnostic tools is a declared priority on the malaria elimination agenda. One promising diagnostic approach is the utilisation of the magnetic properties of hemozoin (HZ) crystals, as it may enable the rapid and quantitative detection of the infection at low cost^5^.

During the course of their erythrocytic cycle the parasites break down the hemoglobin of the host cell and sequester its heme content into HZ crystals, which are distinguishing features of all blood-stage human *Plasmodium* infections^6^. During the formation of these highly inert crystals in the digestive vacuole of the parasites, heme groups are dimerized through iron-carboxylate links and their three dimensional structure is stabilized via hydrogen bonds^7^. HZ has a low-symmetry triclinic crystal structure and the individual crystallites have an elongated rod-like shape, and show species-specific morphological variations^8^. Their structure and morphology has been studied extensively, with special emphasis on their in situ formation and orientation in the digestive vacuole of the parasite^9–11^; their role in antimalarial drug action^12–14^ and their pathogenic and immune-modulatory effects^15–17^. These studies, focusing on fundamental biological aspects of HZ, mostly relied on low-throughput laboratory-grade real-space imaging techniques such as X-ray microscopy, soft X-ray tomography, electron microscopy or spinning disk confocal microscopy^8,10,18^.

As it has been described previously, the anisotropic magnetic and optical properties of HZ can be utilized for its simple, yet highly sensitive detection and quantification in samples derived from cell cultures, animal models and malaria patients^5,19,20^. The quantification of HZ in blood samples of suspected malaria patients has the benefit of providing a quantitative parameter that can aid the assessment of disease severity, in contrast to RDTs for example, which only give a qualitative diagnostic result.

Furthermore, we have demonstrated that the rotating-crystal magneto-optical detection (RMOD) developed by our research group, is an efficient tool for the assessment of drug susceptibility of *P. falciparum* cell cultures via the quantification of their HZ production with high temporal resolution^21,22^. All former studies using the RMOD quantified the parasite growth and changes in parasitemia by the measurement of the overall HZ concentration produced by the parasites, without investigating the size distribution of HZ crystals. In the current study we demonstrate a fundamentally new aspect of the RMOD, namely that it can also yield information about the temporal variation of HZ crystal size during the maturation of malaria parasites.

The principle of size-selective detection of HZ crystals is rooted in their size-dependent dynamics in suspensions exposed to rotating magnetic fields^23^. Since the HZ crystals move freely in suspensions, they can be coaligned by static magnetic fields. Their coaligned ensemble exhibits macroscopic birefringence and dichroism, hence changes the polarisation of the transmitted light beam^24^. The magnitude of this magneto-optical (MO) signal is linearly proportional to the concentration of HZ over a wide concentration range^23^. Importantly, when the magnetic field is rotated, leading to a collective precession of the HZ crystals in the suspension, the MO signal shows a characteristic dependence on the rotational frequency of the magnetic field. Namely, it decreases monotonically with increasing frequency and eventually vanishes at high frequencies when the rotating magnetic field cannot effectively drive the precession of the crystals. Since the magnetic torque is proportional to the volume of the individual crystallites, while the damping effect of the fluid viscosity is proportional to their surface area, it is reasonable to expect that the size of the crystals affects their dynamics and, in turn, influences the frequency-dependence of the MO signal. (For more details on the magnetically driven dynamics of the crystals see ref. Butykai et al*.*).

## Results

### Size distribution of synthetic HZ investigated via scanning electron microscopy and RMOD

According to our previous findings, the frequency-dependence of the MO signal of synthetic HZ suspensions changes by altering the viscosity of the solvent^23^, indicative of a possible crystal-size dependence of the MO signal. The first objective of the current study was to directly test this assumption using multiple suspensions of synthetic HZ crystals with distinct crystal-size distributions. For this purpose, aqueous HZ suspensions with different crystal populations were prepared by the centrifugation of a stock suspension with various centrifugation speeds, and the MO signals of the samples were recorded as the function of the frequency of the rotating magnetic field, which varied in the range of 3–83 Hertz. The crystal-size distribution of the samples was determined directly by scanning electron microscopy (SEM).

**Figure 1:**
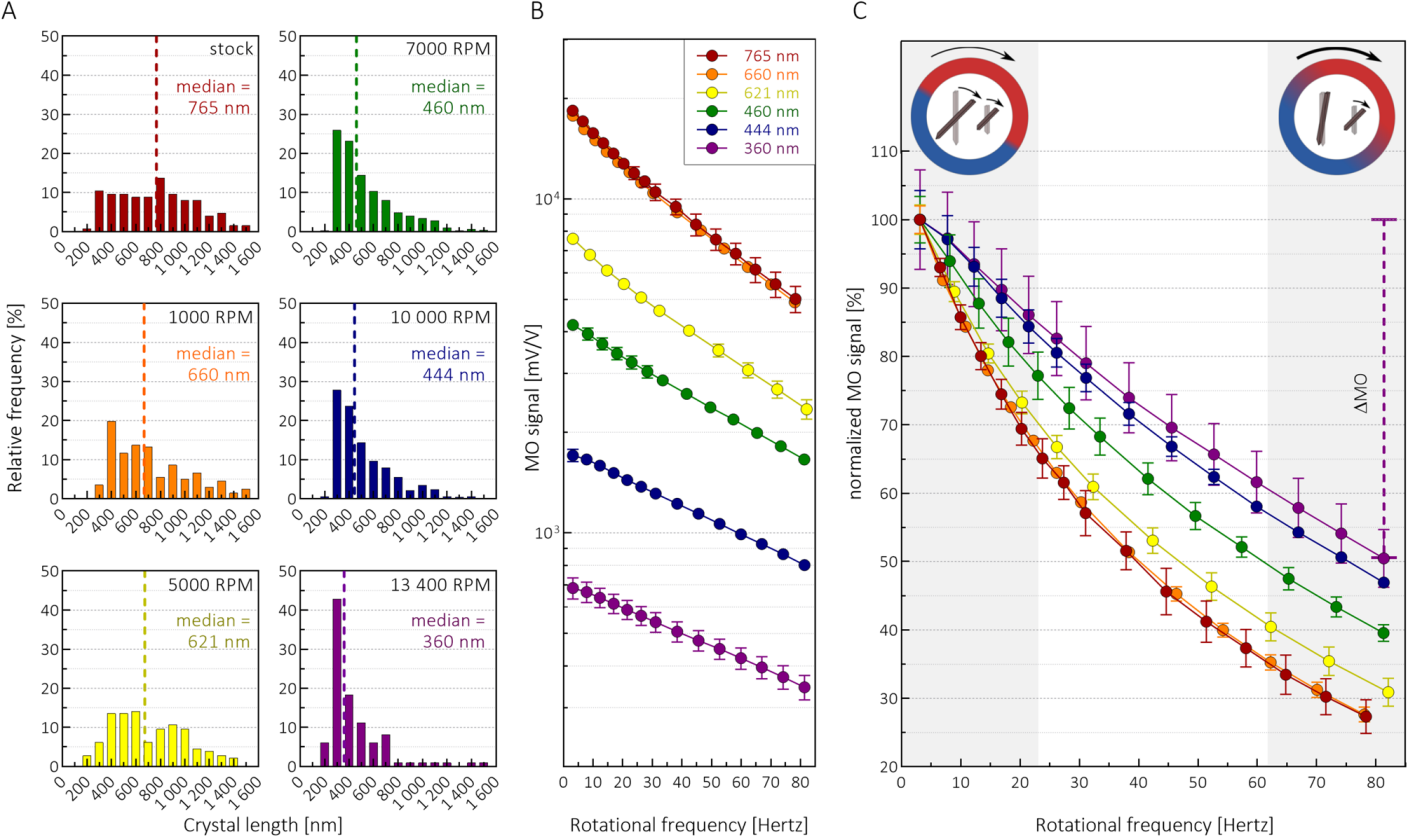
SEM and frequency-dependent magneto-optical measurements on synthetic HZ suspensions. Panel (**A**) The length distribution and median length of the crystals within six synthetic HZ suspensions prepared by centrifugation with increasing centrifugation speeds (indicated in the top right corner of the graphs together with the median length of the crystal populations). Panel (**B**) MO signals of the samples presented in panel A as a function of the rotational frequency of the magnetic field. The differences in the overall signal magnitudes of the different samples is due to the changes in HZ concentration between the suspensions—a consequence of the preparation process (for details see Methods). The legend indicates the median crystal length of the samples. Please note that the y-scale is logarithmic. Panel (**C**) The curves shown in Panel B divided by their MO values at 3 Hertz, i.e., the normalized MO curves as the function of the rotational frequency of the magnetic field. The color-coding of the curves corresponds to the coding used in Panel B. Please note that the frequency-dependence becomes more-and-more pronounced as the median crystal size increases. The error bars represent the standard deviation of measurements on triplicates. The schematics on the top indicate the frequency-dependent dynamics of HZ crystals. While at low rotational speeds of the magnetic field both small and large crystals precess, at higher frequencies only small crystals keep rotating and contributing to the magneto-optical signal.

As Fig. 1A illustrates, the characteristic length distribution and the median length of the crystallites in the supernatants used for the study decrease with increasing centrifugation speeds, as expected. The overall magnitude of the MO signal (Fig. 1B), which is linearly proportional to the crystal concentration at a given rotational frequency, also decreases, since the crystal concentration is reduced by the centrifugation process.

In order to obtain a signal that exclusively reflects the crystal size distribution, and is not influenced by the overall quantity of HZ, we divided the MO curves presented in Fig. 1B by their values at the lowest rotational frequency of the magnetic field, i.e., we normalized all curves at 3 Hertz. The frequency-dependence of these normalized MO curves (Fig. 1C) carries information about the characteristic size distribution of HZ: As the median crystal length gradually decreases from 765 (95% CI: 702–814) nm to 360 (95% CI: 393–490) nm, the decrease observed in the corresponding normalized MO curves between 3 and 83 Hz is also reduced from 73 ± 2 to 50 ± 4%. Regarding the dynamics of the crystals, the stronger frequency-dependence of the normalized MO curves in the case of larger crystal populations indicates that larger crystals stop precessing around the magnetic field at lower frequencies than smaller ones, hence their contribution to the MO signal vanishes already at lower frequencies.

### Changes in HZ size during the maturation of cell cultures

**Figure 2:**
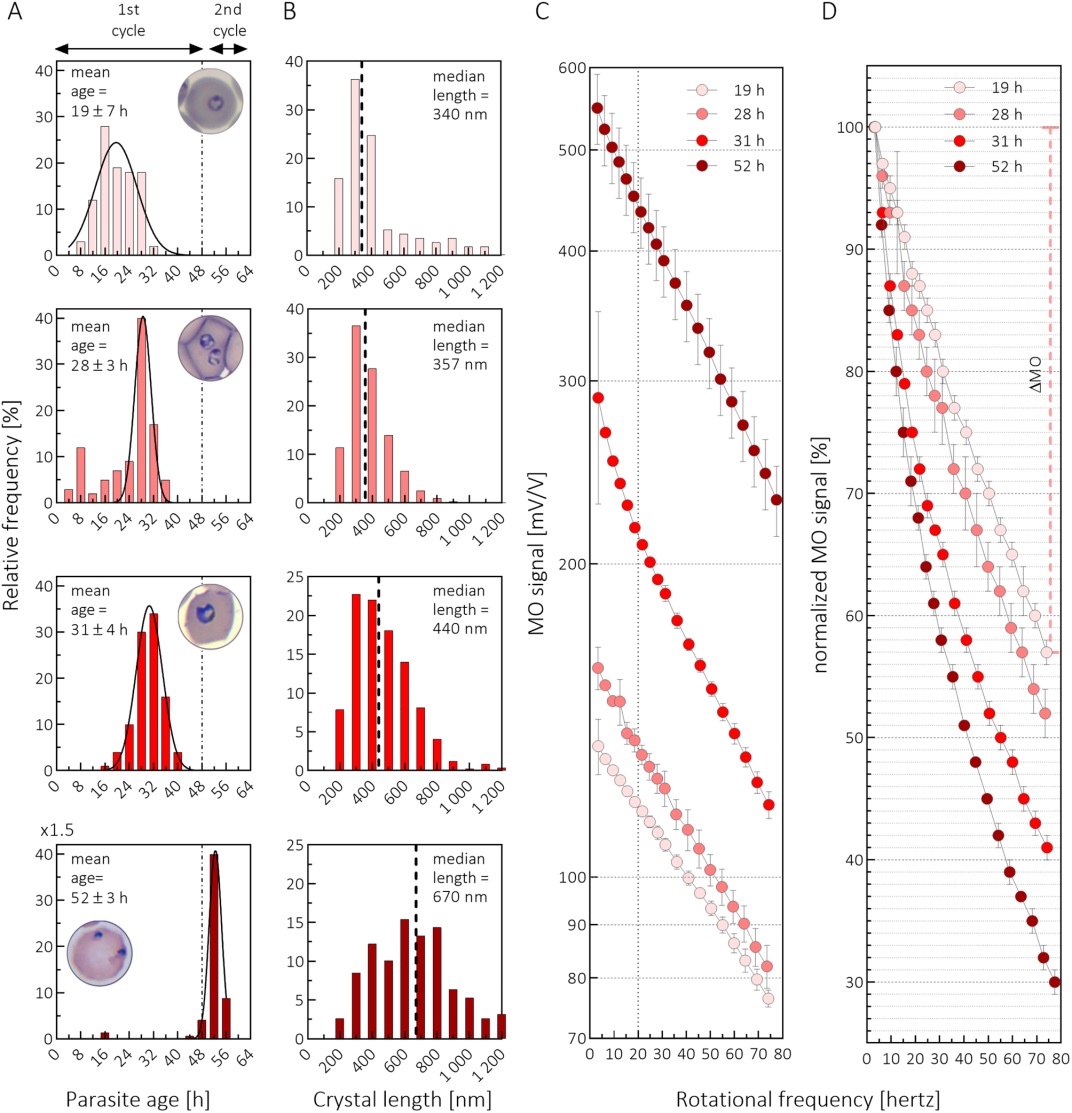
SEM and frequency-dependent magneto-optical measurements on HZ crystals extracted from *Plasmodium falciparum* cell cultures. Panel (**A**) The age distribution of the parasites in four in vitro cell cultures as determined by light microscopic (LM) analysis of thin blood smears (for details see Methods). The insets show LM images of parasites typically found in the respective cultures. Panel (**B**) The length distribution of HZ crystals extracted from the corresponding cultures as determined by SEM analysis. Panel (**C**) The MO signals of the cultures presented in panel A as a function of the rotation speed of the magnetic field. Panel (**D**) The frequency-dependent MO curves divided by their value at 3 Hertz, i.e., the normalized MO curves. Data marked with the same colour correspond to LM, SEM and MO measurements performed on the same parasite culture. In case of Panels C and D the error bars represent the standard deviation of measurements performed on triplicate samples derived from the corresponding culture presented in Panel A.

In order to test whether the RMOD is similarly efficient in characterizing the crystal size distribution of HZ originating from biological samples, we harvested synchronized *P. falciparum* cell cultures at various time points of their intraerythrocytic maturation. We again subjected the samples to MO measurements and extracted the HZ crystals to directly determine their size distribution by SEM analysis. Figure 2 displays four representative examples from these series of experiments. Figure 2B,C reveal that both the HZ crystal size and overall quantity increases substantially as the parasites mature from early-trophozoites with a mean age of 19 h to late-schizonts with a mean age of 48 + 4 h. (Please note that although the latter culture was harvested after entering the second erythrocytic cycle around 48 h, the HZ crystals present in the sample were predominantly produced by late-schizonts of the first cycle.) The median crystal size increases from 340 (95% CI: 316–385) nm to 670 (95% CI: 620–714) nm during this period, while the amount of HZ crystals increases by a factor of four. Furthermore, the proportion of crystals with lengths above 800 nm significantly increases by the end of the erythrocytic cycle. While their proportion in the crystal populations produced by trophozoites typically stays below 10%, by the end of the cycle they constitute almost 40% of the observed HZ crystals.

The normalized MO curves (Fig. 2D) show the same tendency as seen in the case of the synthetic HZ measurements. As the median crystal size increases, the difference between the low and high frequency values of the normalized MO curves also increases. Accordingly, these four examples indicate that there is a direct correspondence between the mean age of the culture, the median crystal length and the frequency dependence of the normalized MO curves. In order to quantify these relationships, we introduce a single parameter for the characterization of the frequency-dependent normalized MO curves: $\Delta MO \left[ \% \right] = \frac{{\left[ {MO\left( {3 Hertz} \right) - MO\left( {70 Hertz} \right)} \right]}}{{MO\left( {3 Hertz} \right)}} = 100\% - \frac{{MO\left( {70 Hertz} \right)}}{{MO\left( {3 Hertz} \right)}}\left[ \% \right].$

**Figure 3:**
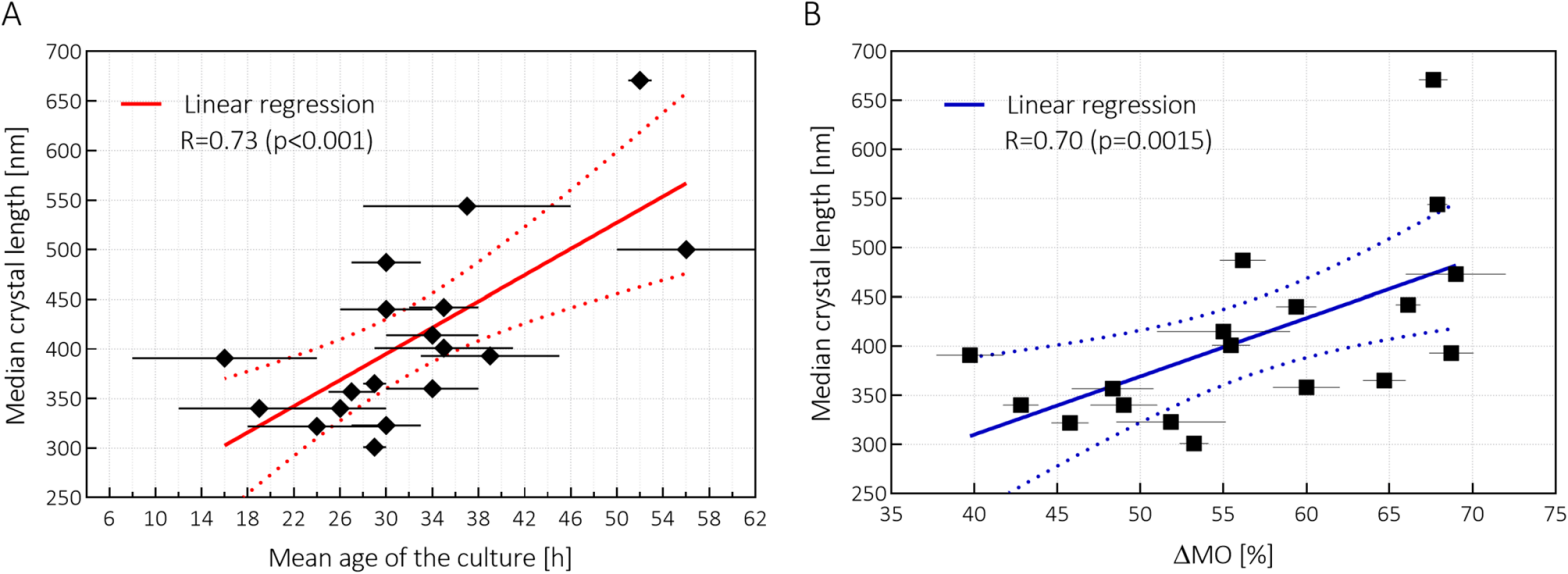
Correlation between the median crystal size, the mean age of the cultures and the ΔMO parameters (which characterize the frequency-dependence of the MO signals). Panel (**A**) The median length of the HZ crystals extracted from eighteen cell cultures as the function of the mean age of the parasite population. The red line represents the linear regression fit of the data, with the Spearman rank correlation coefficient also indicated. Panel (**B**) The median length of the HZ crystals extracted from the cell cultures presented in panel A as the function of the corresponding ΔMO values. The blue line represents the linear regression fit of the data, with the Spearman rank correlation coefficient also indicated.

**Figure 4:**
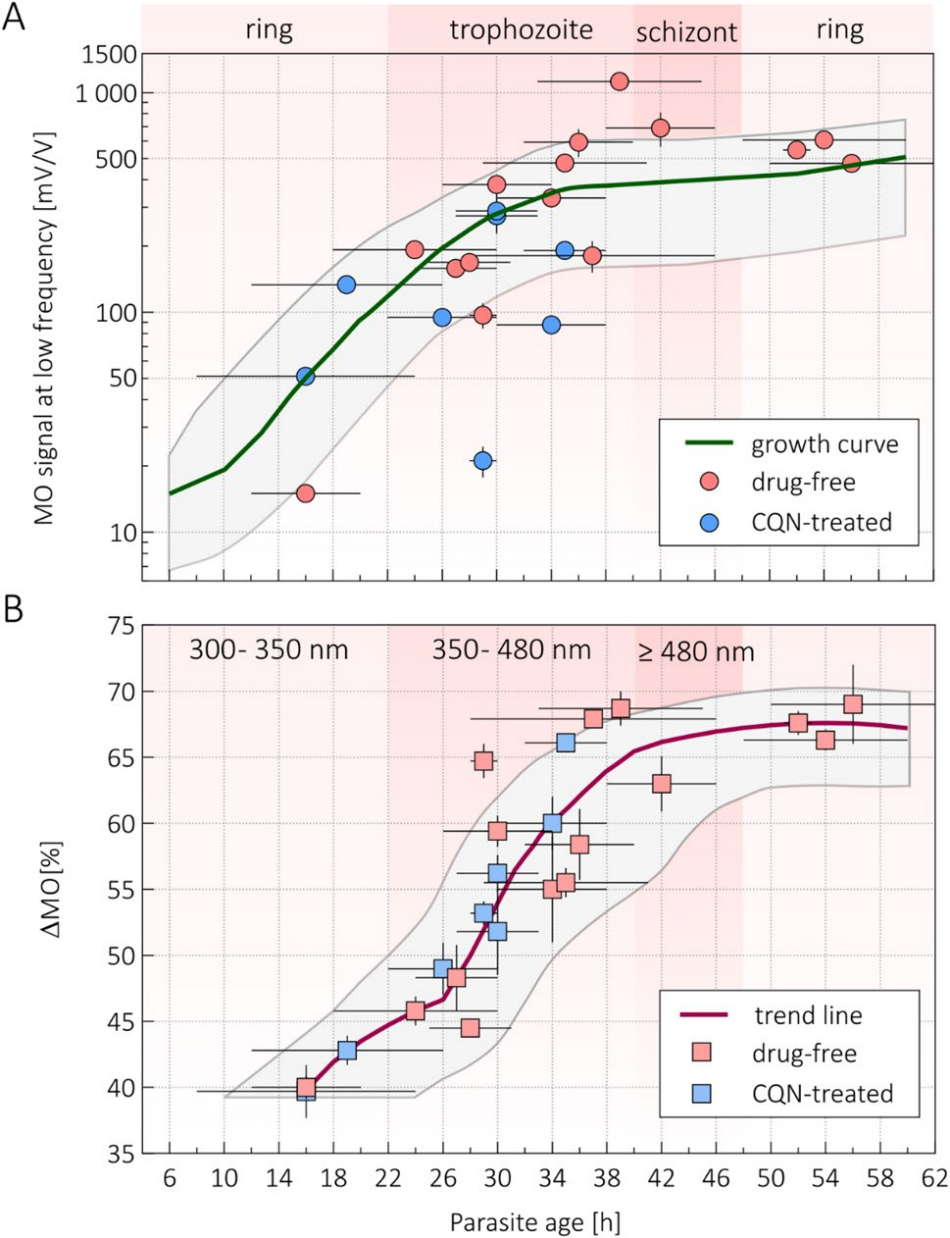
The HZ production characteristics of *P. falciparum* cell cultures monitored by RMOD measurements. Panel (**A**) The quantity of HZ produced by *P. falciparum* cultures, as represented by their MO values at 3 Hertz, versus their estimated mean erythrocytic age. The green ‘growth curve’ was established previously via the RMOD-based HZ quantification of several independent maturation assays^22^. The individual data points show the low-frequency MO values of cell cultures investigated in the current study. Pink and blue data points mark cultures incubated drug-free and with 50 nM chloroquine, respectively. Please note that the vertical scale is logarithmic. Panel (**B**) ΔMO versus the estimated erythrocytic age of the cultures investigated in the current study. The purple curve is the average of these individual data points. The color-coding corresponds to that of Panel A. The median crystal sizes observed in the beginning and end of the major maturation stages is also indicated in the top row of the graph.

As Fig. 3A illustrates, the Spearman correlation coefficient between the mean parasite age and the median crystal size—determined by SEM analysis on HZ extracted from eighteen independent *P. falciparum* cultures between the ages of 14 h and (48 + 8) h—can be regarded as moderately high with a coefficient of $R=0.73 \left(p<0.001\right)$. Furthermore, the same coefficient between the median crystal size and the corresponding $\Delta MO$ parameters is also significant with a value of $R=0.70 (p=0.0015)$ as seen in Fig. 3B. Our analysis reveals that the median length of crystals produced by early-trophozoites (approx. 18 h old) is around 315 ± 60 nm and it increases gradually to 550 ± 80 nm by the end of erythrocytic cycle. The $\Delta MO$ parameters also increase from 40 ± 4 to 70 ± 5% during this period confirming that the increase in the average crystal length of biological samples is reflected by the frequency-dependent MO curves. Accordingly, Fig. 3B shows a key result of this work, namely that not only the concentration but also the size distribution of biological HZ crystals can be investigated by RMOD. Please note that among the three quantities, that are the median crystal size, the mean parasite age and $\Delta MO$, the first has the largest error due to the limited number of crystals which could be extracted for the SEM analysis. Correspondingly, the direct correlation between the mean parasite age and $\Delta MO$ is considerably better than their correlations with the median crystal size, as seen later in Fig. 4B.

In order to further elaborate on the connection between the frequency-dependent MO signals and parasite maturation, we also investigated synchronized *P. falciparum* cell cultures incubated with chloroquine at 50 nM concentration for 14 or 34 h. In Fig. 4, we summarize the results obtained for these drug-treated samples together with those shown for untreated cultures in Fig. 3. The overall amount of HZ produced by the parasites, as quantified by their MO value at 3 Hertz, versus their mean age is in good agreement with our previously established average HZ production curve (‘growth curve’ in Fig. 4A)^22^. This indicates that the cultures matured as expected and further supports the reliability of our HZ quantification method. As Fig. 4A reveals, there is a detectable HZ production already at the mid-ring stages (10–18 h) followed by a considerable increase until the beginning of the schizont stage when the crystal formation is halted until the mid-ring period of the next generation.

As the important new insight gained here, the $\Delta MO$ parameter also shows a strong correlation with the mean age of the cultures (Spearman R = 0.90; *p* < 0.001) and follows a characteristic trend. After a moderate increase in the late-ring and young-trophozoite period (14–26 h) a sharp increase can be observed during the mid-trophozoite and early-schizont stages (26–40 h) followed by a plateau phase at the end of the cycle. The temporal comparison of HZ accumulation and crystal growth reveals that, while the most intense HZ production happens between 18 and 34 h, the most pronounced phase of crystal growth only occurs later, in the 26–40 h time period.

Concerning the drug-treated parasite cultures, their MO values at 3 Hertz as well as their $\Delta MO$ values follow the trend of the drug-free samples. This observation may seem counterintuitive, given that chloroquine is known to be a HZ inhibitor and in this study it was applied in 50 nM concentration, that is above the IC-50 value for 3d7 strain^25^. However, one has to note that during the microscopic evaluation of the mean age of the populations, only the surviving parasites with healthy morphology were considered in the age classification. Correspondingly, the dead parasites, being a remarkable fraction of the population, are not included when determining the mean age of the population. Their contribution to the HZ production is expected to be small, since the cultures treated by chloroquine were synchronized cultures with ring-stage parasites. Therefore, our analysis, investigating the correlation between median crystal size, mean parasite age and $\Delta MO$ under drug pressure, can only account for secondary drug effects. Namely, for “healthy-looking” parasites the maturation can be delayed due to drug-induced dormancy^26^. The fact, that the dependence of the MO values at 3 Hertz as well as the $\Delta MO$ values on the mean parasite age follow very similar trends for drug treated and untreated cultures, implies that the delay in maturation due to drug inhibition is reflected in the parasite morphology and the produced HZ content (crystal size and amount) in the same fashion. In other words, the HZ crystal size is characteristic to the maturation stage, irrespective if the maturation of the parasite was delayed or not due to drug exposure.

## Discussion

Since HZ crystallization is a vital process of the intraerythrocytic development of the malaria parasites—with the exception of few genetically modified laboratory strains^27,28^—the means of HZ production by Plasmodia have been studied quite extensively with special emphasis on its role in the mechanism of action of quinoline compounds^12,14,29^. However, to the best of our knowledge, no statistical analysis of the crystal size distribution of HZ extracted from bulk in vitro cultures has been published before. Our SEM-based size distribution analysis can be regarded as complementary information to the results of Gligorijevic and co-workers and Kapishnikov and co-workers who quantified the overall amount of crystalline HZ and the size and shape of individual crystallites on a cellular level, respectively^10,11,30^. The study of Gligorijevic et al*.*, using spinning disc confocal microscopy, found that the volume of HZ within the parasites plateaus at approximately 0.6 µm^3^ around the late trophozoite to early schizont boundary. The studies of Kapishnikov et al*.* performed via soft X-ray tomography on a smaller sample size detected slightly lesser amounts. They found the total volume of HZ to be approx. 0.17 ± 0.14 µm^3^ and 0.42 ± 0.17 µm^3^ in the digestive vacuoles of trophozoites and schizonts, respectively. Our statistical analysis of hundreds of extracted crystals suggests that the length of an average crystal in the mid-trophozoite stage (~ 28 h old parasite) is 380 ± 30 nm and it increases to 550 ± 80 nm for late-stage schizonts (~ 46 h old parasite). Considering these lengths and assuming that the edges of the approximately rectangular HZ crystallites have relative lengths of 1:1:3.5 (based on ref. Buller et al*.* and our SEM observations)^31^, the volume of a typical HZ crystal in the trophozoite and schizont stage is (4.5 ± 1)*10^–3^ µm^3^ and (13.6 ± 5.9)*10^–3^ µm^3^, respectively. The comparison of these data to the overall HZ volumes found by Kapishnikov et al*.* yields that the number of individual crystallites in the digestive vacuoles of trophozoites is 38 ± 33, while the number of crystals in schizonts is practically the same within the margin of error, 31 ± 18. The study of Kapishnikov et al*.* also quantified the number of crystallites in the vacuoles of a few selected parasites and estimated their average number to be 40 ± 20^11^. The good agreement between this number and the findings of the current study suggests that (1) the total volume of HZ per cell is approximately doubled between the mid-trophozoite and late-schizont development reaching a maximum of approx. 0.42 µm^3^; (2) this increase is not due to the nucleation of new crystals since their number does not change significantly during this period; but rather because of (3) the continuous growth of the ones formed in the previous stages of parasitic development.

While the above listed techniques enable insight into the intricate details of HZ formation, they require sophisticated instrumentation and relatively burdensome and time consuming sample preparation. In the current study we aimed to demonstrate that the RMOD provides a useful alternative for the assessment of the amount of HZ crystals and their size distribution. We found a significant correlation between the frequency-dependent MO signals and the mean age of synchronized *P. falciparum* cultures due to the changes in crystal size during maturation. Furthermore, the parallel quantification of HZ concentration and crystal size distribution via RMOD enabled the simple assertion of the temporal shift between the most intense phases of HZ accumulation and crystal elongation.

In our previous study we demonstrated that the quantification of the overall HZ content of a synchronized in vitro* Plasmodium* culture is an effective tool to test the potency of antimalarial compounds^22^. The amount of HZ produced by a well-synchronized culture, however, depends on the parasitemia of the sample. Since the $\Delta MO$ parameters yield information about the mean crystal length independently of the total amount of HZ, they can characterize the mean age of the parasite population of the sample independently of its parasitemia. Accordingly, such measurements can aid the monitoring and the quantitative comparison of parasite growth even for samples with different parasitemia levels, for example in case of ex vivo patient samples incubated with antimalarial compounds. Clearly, the assessment of parasitemia can be carried out easily using light microscopy, but the quantitative comparison of population mean ages is burdensome, especially within one erythrocytic cycle. We believe that besides the several sophisticated methods used for the assessment of antimalarial drug activity, e.g., ELISA, hypoxanthine assay, flow cytometry, PCR, etc., the RMOD provides a sensitive and cost-efficient alternative. There are, however, certain limitations of the RMOD-based monitoring of parasite maturation that need to be acknowledged. In case of in vitro experiments thorough washing is needed to avoid contamination with crystals from previous cycles, and the throughput of the method needs to be increased to be able to process several dozens of samples within an hour.

Besides the applicability of RMOD as a laboratory tool, the current study has implications for the hemozoin-based in-field malaria diagnostics as well. As mentioned above, the very simple, yet quantitative assessment of parasite maturation could be especially useful in resource limited settings for carrying out ex vivo drug susceptibility assays to aid therapy and monitoring the spread of drug resistance.

Furthermore, since parasite stage distribution differs significantly in *P. falciparum* and *P. vivax* infections, we hypothesise that the simultaneous magnitude and frequency dependent measurements can be used to predict if an infection is either *P. falciparum* or *P. vivax*^8,32,33^. While we do not expect this to yield a perfect dichotomisation of species, it could provide at least as good accuracy as the current RDTs. However, further large-scale field studies involving both species are necessary to test this hypothesis.

The RMOD utilizes two physical properties of the HZ crystals for the quantification of their concentration and size distribution, their magnetic anisotropy and their optical anisotropy. The former facilitates the co-alignment of the crystals in suspension, while the latter produces optical birefringence/dichroism for the co-aligned ensemble. As described earlier, when the magnetic field is rotated, the crystals can or cannot follow it depending on their size. Thus, the dependence of the MO signal on the rotational frequency of the magnet makes the method size selective. Although we have applied this principle so far only for HZ-based malaria diagnostics, we believe that the RMOD principle can be adapted for the detection and volumetric characterization of other micro- and nanometer-sized particles as well, if they fulfil the two criteria mentioned above. We expect that the technique is not limited to paramagnetic materials with anisotropic magnetic susceptibility, but it is also applicable to magnetically isotropic paramagnetic and even diamagnetic particles with shape anisotropy, as pointed out in ref. Rikken et al.^34^ Accordingly, the method may be applicable e.g., to non-spherical microbeads with paramagnetic iron-oxide cores and even diamagnetic non-spherical cells. In summary, the scope of this rapid and easy-to-use technique may cover the high-sensitivity concentration measurement and size-assessment of various micro- and nanometer-sized particles within biomedical research and beyond.

## Materials and methods

### Synthetic HZ measurements

All chemicals and reagents listed in the Materials and Methods section were purchased from VWR International (Radnor, PA, USA) unless stated otherwise.

The HZ crystals were synthetized following the aqueous acid-catalyzed method described by M. Jaramillo and co-workers^35^. Briefly, 500 mg of hemin [iron(III)(protoporphyrin-IX)Cl, purchased from Sigma-Aldrich, Merck Group, Darmstadt, Germany] was dissolved in NaOH (0.1 M, 100 ml) with the dropwise addition of propionic acid adjusting a pH value of approximately 4. After annealing the mixture for 18 h at 70 °C, the solid particles were separated and washed with NaHCO3 (0.1 M), MilliQ water and MeOH, multiple times, alternately – as prescribed by the authors. Then the sample was dried in a vacuum oven overnight over phosphorus pentoxide.

For the formation of HZ suspensions with different crystal size populations, first a stock of aqueous HZ suspension was prepared with a concentration of 40 µg/ml, and subjected to ultrasonication for 3 × 10 min, alternated by short vortexing, to fully disperse the individual crystallites. Several 1.5 ml Eppendorf tubes were filled with 1 ml of the homogenous stock suspension, and the tubes were pairwise subjected to centrifugation with different spinning speeds (as indicated in Fig. 1A) for 3 min in an Eppendorf MiniSpin ultracentrifuge. After centrifugation 0.5 ml of the supernatants from both tubes were carefully removed and set aside for SEM and RMOD measurements.

For the RMOD measurements two optical sample holders were filled from each of the duplicate supernatants and measured without further processing. Accordingly, one MO curve presented in Fig. 1B. is the average of four independent RMOD measurements.

For SEM imaging a few μl of the removed supernatants were dropped on 8 × 8 mm oxide-free silicon wafers and left to dry overnight under ambient conditions.

### RMOD measurements

The scheme of the RMOD setup, as well as the underlying physical principles of the detection, are described in detail in our former studies^21,23^. Briefly, the detection of hemozoin is based on its paramagnetic and dichroic properties. A strong, rotating magnetic field is used to induce the collective spinning of the crystals in the liquid medium, which results in periodic changes in the transmitted intensity of a polarized laser light. The amplitude of the intensity oscillations divided by the average transmission is defined as the magneto-optical (MO) signal measured in units of mV/V. During an MO measurement a cylindrical sample holder with a volume of 220 μl is filled with the liquid sample (e.g., synthetic HZ suspension, diluted and hemolysed blood sample, etc.) and inserted into the bore of a Halbach magnetic ring. The ring is rotated by a DC motor in the 3–83 Hertz frequency range with arbitrary pre-defined frequency-steps. At each frequency value the transmitted intensity is collected and analysed for a few seconds. Accordingly, the recording and analysis of a magneto-optical curve takes approximately 3–5 min depending on the desired frequency-resolution.

### *Plasmodium falciparum* cell cultures

*Plasmodium falciparum* 3D7 strain was grown in A + erythrocytes (Hungarian National Blood Transfusion Service, Budapest, Hungary) not older than a month in complete malaria culture medium (according to the recommendations of the Malaria Research and Reference Reagent Resource Center (MR4, Bei Resources))^36^. Cultures were maintained at 5% hematocrit, 37 °C in an atmosphere of 5% O_2_, 5% CO_2_ and 90% N_2_. Cultures were synchronized regularly by applying the sorbitol and density gradient centrifugation methods^36^. Briefly, for the sorbitol method, cultures of at least 2% parasitemia and mainly ring stage parasites were incubated in 5% sorbitol (Sigma-Aldrich, Merck Group, Darmstadt, Germany) solution for 10 min at 37 °C. After washing, parasites were cultured as described above. For the density gradient centrifugation, cultures containing mostly schizont stage parasites were centrifuged with 70% Percoll (Sigma-Aldrich, Merck Group, Darmstadt, Germany) in order to separate the schizont stage parasites. The aforementioned techniques were used in combination throughout several cycles in order to achieve well-synchronized cultures.

In order to assess the stage distribution of the cultures with high temporal resolution, an intraerythrocytic parasite maturation guide was created from thin smear microscopy images representative of our assays (Supporting Information Fig. S1). The absolute ages of the parasites in the guide were determined by comparing them to *Plasmodium falciparum* morphologies available in the literature^37–39^. For each cell culture at least 100 parasites were categorized based on the maturation guide by two microcopists to obtain the stage distribution histograms and the mean age of the cultures at the time of sampling.

### Culture sample preparation for RMOD and SEM measurements

Ring stage cultures were synchronized and washed three times in RPMI 1640 (Biowest, Nuaillé, France) in order to remove HZ produced in previous parasite cycles. Cultures were set to 5% hematocrit and parasitemia values between 1 and 3%, and incubated in cell culture flasks. At designated time points the cell cultures were thoroughly homogenized after the preparation of thin blood smears, and 3X160 μl of the cell suspension was set aside for MO measurements. The remaining culture was either incubated as described above for further sampling, or it was used for HZ extraction. In case of the drug experiments the ring stage culture was separated into two halves at the beginning of the assay and half of the culture was incubated with chloroquine at a concentration of 50 nM, and the other half was maintained drug-free. After 14 and 34 h of incubation both the drug-treated and the drug-free control cultures were sampled for LM analysis and MO measurements. Altogether three independent drug assays were performed with two sampling times, i.e., six drug-treated and six drug-free control cultures were analysed.

For the RMOD measurements each aliquot of the 160 μl cell suspension was directly diluted 3X with a cell lysis buffer (13 mM of NaOH and 0.03% Triton X-100 in MilliQ water), mixed thoroughly by pipetting and gentle vortexing. After 5 min on room temperature the samples were carefully homogenized, filled directly into optical sample holders and subjected to RMOD analysis.

### HZ extraction from cell cultures

After sampling for MO measurements and LM analysis, the remaining cultures with a haematocrit of 5% and a volume of approx. 5 ml were transferred into 15 ml Falcon tubes and centrifuged (4000 RPM, 10 min). The supernatant was removed and the parasitized red blood cells were hemolysed with 6 ml of a lysis solution consisting of 0.03 vol% Triton X-100 in 6 mM NaOH. The lysate was distributed into six 1.5 ml Eppendorf tubes and centrifuged in an Eppendorf MiniSpin ultracentrifuge (13 000 RPM, 10 min). Approx. 0.7 ml of the supernatant was removed and the reddish pellets were washed with 1 ml distilled water (DW). The washing step included 10 min of sonication, vortexing and centrifugation as described above. After this step the size of the pellets reduced substantially and the supernatant was almost transparent. After careful removal of approx. 1 ml supernatant, the pellets were washed twice with sodium dodecyl sulfate (SDS, 2 vol%) and twice with DW. The remaining small, brownish pellets were merged, mixed with approx. 1 ml of urea (6 M), sonicated for 10 min and incubated in a shaker for 3 h at 37 °C. After incubation the samples were centrifuged, the supernatant was removed and the pellets were washed once with DW, twice with SDS (2 vol%) and finally twice with DW. After the last washing step, the very small brown pellets were re-suspended in 80 μl of MilliQ water and sonicated for 15 min. The samples were thoroughly mixed and small droplets of the suspensions containing the HZ crystals were dropped on 8 × 8 mm oxide-free silicon wafers and left to dry overnight under ambient conditions.

### SEM imaging and analysis

SEM imaging of synthetic HZ was performed on an Thermo Scientific Scios 2 electron microscope, using an Everhart–Thornley detector in secondary electron mode with the following settings: accelerating voltage 5 kV; current 1.6 nA and working distance of 7.1 mm. The corners of the substrates were connected to the metallic sample holder using a piece of conductive carbon tape in order to reduce charging effects. SEM images were acquired at random locations of the substrates with magnifications between 500 × and 8000 × and the collected scans were examined by visual inspection to find optimal areas for size analysis. For size distribution analysis, images with a magnification of 2500 × and resolution of 1530 × 1094 pixel were used from multiple droplets and areas of the corresponding samples.

All images were pre-processed with ImageJ (National Institute of Health, USA, http://imagej.nih.gov/ij). After calibration of the image scale, HZ-like particles were separated from the background based on contrast by selecting an optimal threshold value. Thereafter, particles with an area above 20 nm2 were filtered by automated image analysis and the remaining selections were scanned by visual inspection to discard particles not consistent with the elongated HZ morphology. The remaining particles were characterised by their maximal Feret diameter. Accordingly, the terms ‘size’ and ‘length’ in case of the synthetic HZ samples refer to this parameter of the identified crystals. Finally, the lower and upper 10% of the obtained length values were discarded. The number of crystals included in the final statistics ranged between 100 and 700 for each histogram presented in Fig. 1. The error of the individual length values is estimated to be 50–60 nm. Typical SEM images and steps of the image analysis process are shown in Supplementary Figs. S2 and S3.

SEM imaging of HZ extracted from cell cultures was performed on a ZEISS Crossbeam 550 electron microscope with HE-SE2 detector, using the following settings: accelerating voltage 5 kV (in some cases 15 kV); current between 5 pA and 4 nA and working distance between 5 and 8.7 mm. The corners of the substrates were connected to the metallic sample holder using a piece of conductive carbon tape in order to reduce charging effects. SEM images were acquired at random locations of the substrates with magnifications ranging between 500 × and 60,000 × and the collected scans were examined by visual inspection to find optimal areas for size analysis. In a few cases we performed energy dispersive X-ray spectroscopy on particles with the most typically observed geometry to obtain information about their iron content and thus confirm that the objects are HZ crystals. For size distribution analysis, images with a magnification of 5000 × and resolution of 16,384 × 12,288 pixel were used from multiple droplets and areas of the corresponding samples.

Due to the small overall number of crystals, the presence of clusters and biological matter, the crystal size analysis was performed manually in the case of the cell culture samples. Namely, particles consistent with HZ morphology were identified by the examiner and lines were drawn along the longitudinal axis of the crystallites. The length of the line segments was evaluated by a built-in algorithm of ImageJ. Accordingly, the terms ‘size’ and ‘length’ in case of the natural HZ samples refer to this parameter of the identified crystals. In order to eliminate operator bias, the examiner was unaware of the name of the sample during analysis. Furthermore, in some cases the images were re-analysed by a second, independent investigator. The number of crystals included in the final statistics ranged between 100 and 700 for each histogram and median presented in Figs. 2 and 3, respectively. The error of the individual length values is estimated to be 10–20 nm. Typical SEM images of extracted crystals are presented in Fig. S4 in the Supplementary information.

### Supplementary Information

Supplementary Information.

## Data Availability

The datasets generated during and/or analysed during the current study are available from the corresponding author on reasonable request.
