# Supplementary material for: Magneto-optical assessment of Plasmodium parasite growth via hemozoin crystal size
Source: Sci Rep. 2024 Jun 21;14:14318. doi: 10.1038/s41598-024-60988-6 (PMC11192761; doi:10.1038/s41598-024-60988-6)
Supplement: Supplementary file 1 — Supplementary Information. [file 41598_2024_60988_MOESM1_ESM.pdf]

Supplementary information

to the article

Magneto-optical assessment of *Plasmodium* parasite growth via hemozoin crystal size

Ágnes Orbán, Jan-Jonas Schumacher, Szilvia Mucza, Ana Strinic, Petra Molnár, Réka Babai, András Halbritter, Beáta G. Vértessy, Stephan Karl, Stephan Krohns and István Kézsmárki

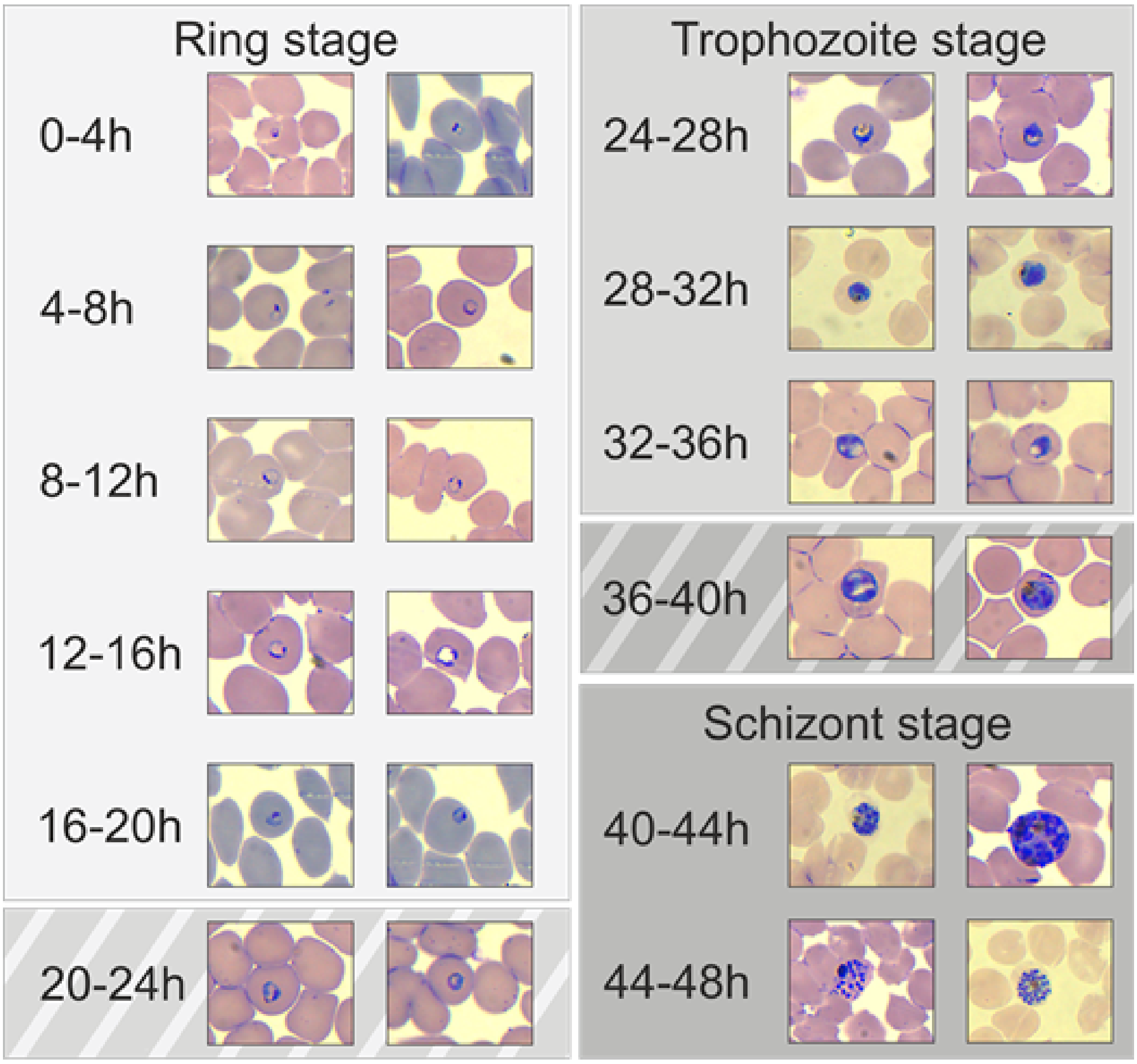

Figure S1. Erythrocytic maturation guide. The images show giemsa-stained thin blood smears of *Plasmodium falciparum* 3D7 strain parasites throughout one erythrocytic cycle *in vitro*.The light microscopy images were taken of parasites typically observed in our cell cultures and their absolute ages were determined by comparison to morphologies available in the literature [1–3].

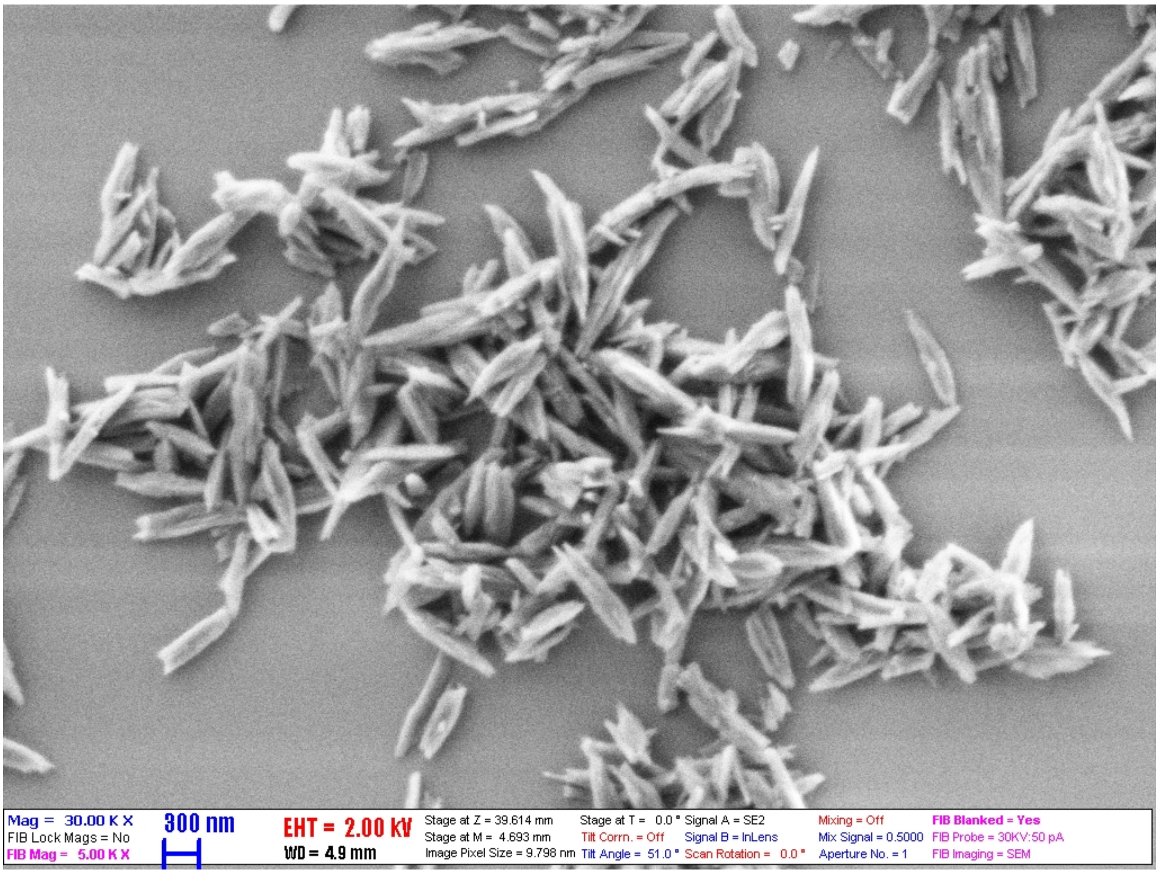

Figure S2. High magnification SEM image of synthetic hemozoin used in the current study. The crystals were synthetized following the aqueous acid-catalyzed method described by M. Jaramillo and co-workers [4]. Please note that this method yields less brick-like crystallites with more tapered ends and less smooth sides than hemozoin formed by *in vitro P. falciparum* parasites.

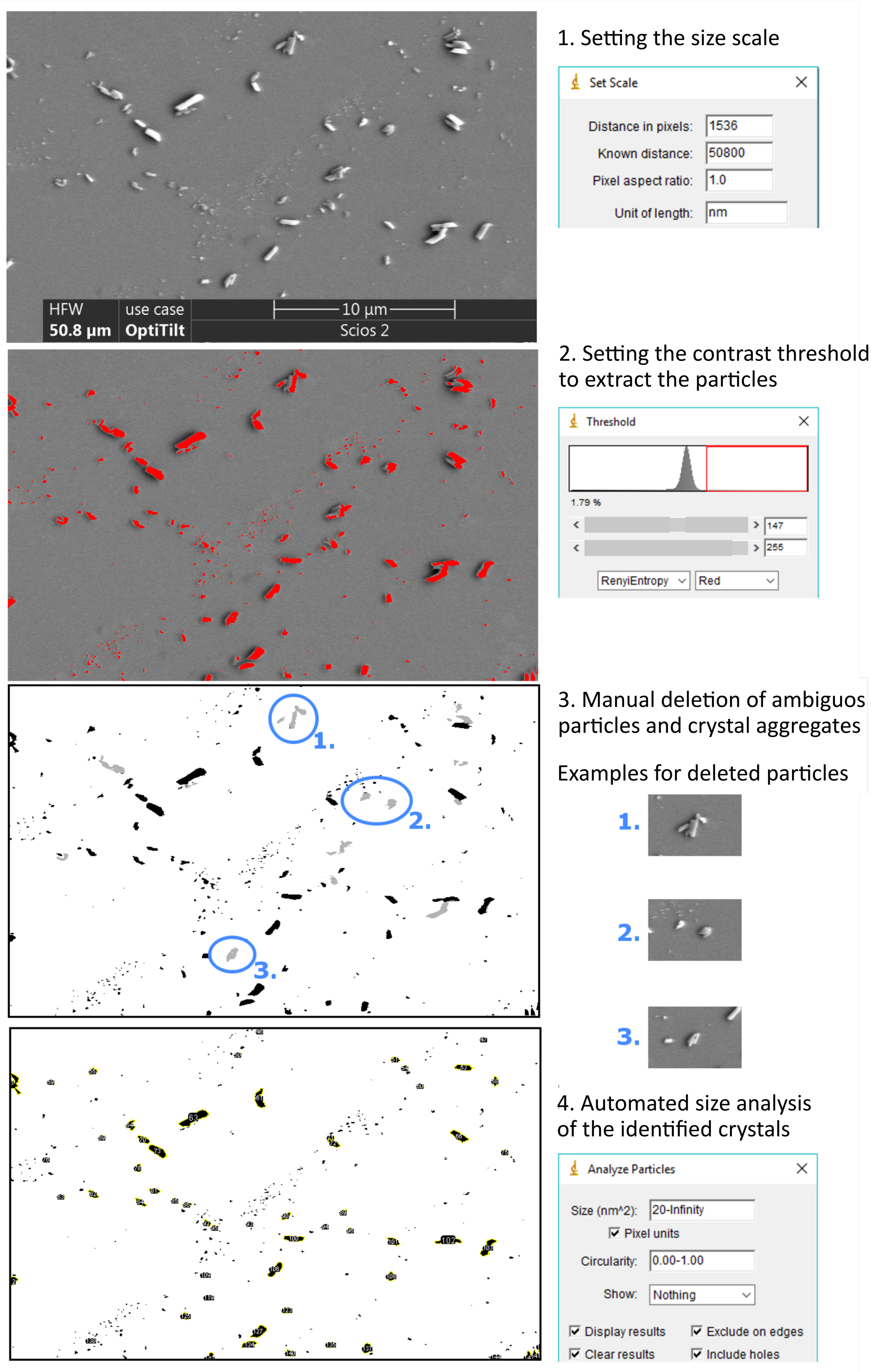

Figure S3. Steps of image analysis for the establishment of size distribution histograms of synthetic HZ samples. All SEM images were evaluated using the ImageJ software. After calibration of the image scale, HZ-like particles were separated from the background based on contrast by selecting an optimal threshold value. Thereafter, particles with an area above 20 nm^2 were filtered by automated image analysis and the remaining selections were scanned by visual inspection to discard particles not consistent with the elongated HZ morphology. The remaining particles were characterised by their maximal Feret diameter.

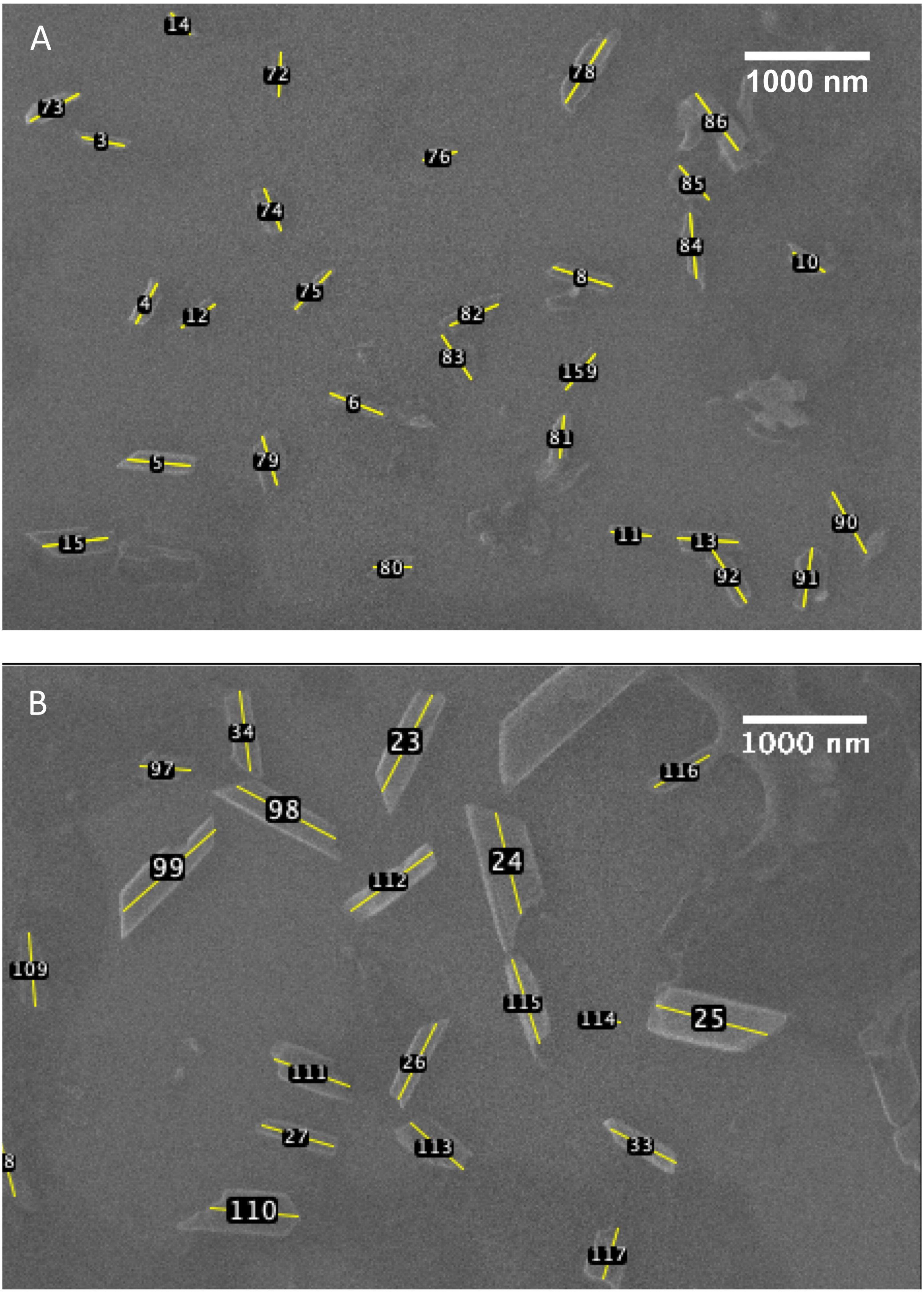

Figure S4. SEM images of HZ extracted from P. falciparum 3D7 parasites. Panel A Crystals extracted from a culture with a mean erythrocytic age of approximately 35 hours, i.e., mid-stage trophozoites. **Panel B** Crystals extracted from a culture with a mean erythrocytic age of approximately 48+4 hours, i.e., late-stage schizonts. The crystals were identified manually. The length of their elongated side is indicated by the yellow segments.

References

1. Kozicki, M., Czepiel, J., Biesiada, G., Nowak, P., *et al.* The ring-stage of Plasmodium falciparum observed in RBCs of hospitalized malaria patients. *Analyst* **140,** 8007–8016 (2015).

2. Grüring, C., Heiber, A., Kruse, F., Ungefehr, J., *et al.* Development and host cell modifications of Plasmodium falciparum blood stages in four dimensions. *Nat Commun* **2,** 165 (2011).

3. Silamut, K., Phu, N. H., Whitty, C., Turner, G. D., *et al.* A quantitative analysis of the microvascular sequestration of malaria parasites in the human brain. *Am J Pathol* **155,** 395–410 (1999).

4. Jaramillo, M., Bellemare, M. J., Martel, C., Shio, M. T., *et al.* Synthetic Plasmodium-like hemozoin activates the immune response: a morphology - function study. *PLoS One* **4,** e6957 (2009).
